# Indirect Methods to Determine the Risk of Damage to the Health of Firefighters and Children Due to Exposure to Smoke Emission from Burning Wood/Coal in a Controlled Environment

**DOI:** 10.3390/ijerph20085607

**Published:** 2023-04-21

**Authors:** Marcelo Sampaio Ocampos, Luana Carolina Santos Leite, Elaine Silva de Pádua Melo, Rita de Cássia Avellaneda Guimarães, Rodrigo Juliano Oliveira, Karine de Cássia Freitas, Priscila Aiko Hiane, Arunachalam Karuppusamy, Valter Aragão do Nascimento

**Affiliations:** 1 Group of Spectroscopy and Bioinformatics Applied to Biodiversity and Health (GEBABS), Graduate Program in Health and Development in the Central-West Region of Brazil, School of Medicine, Federal University of Mato Grosso do Sul, Campo Grande 79079-900, Brazil; 2 Graduate Program in Health and Development in the Central-West Region of Brazil, Federal University of Mato Grosso do Sul, Campo Grande 79079-900, Brazil; 3 Center for Studies in Stem Cells, Cell Therapy and Genetic Toxicology (CeTroGen), School of Medicine, Federal University of Mato Grosso do Sul, Campo Grande 79079-900, Brazil

**Keywords:** heavy metals, metalloids, health risks, fire smoke exposure, firefighters

## Abstract

People are constantly exposed to particulate matter and chemicals released during fires. However, there are still few studies on gas and particulate emissions related to exposure to burning firewood and charcoal during forest fires, making it difficult to understand the effects on the health of the population. The objective of this study was to quantify the metal(loid)s present in the smoke from wood and charcoal fires through the deposition of metals in beef topside and pork loin, considering the routes of skin exposure, inhalation, and ingestion, contributing to the understanding of metals in the increase of the risks of cancer and mortality associated with firefighting and children. The concentrations of metals [aluminum (Al), chromium (Cr), copper (Cu), iron (Fe), magnesium (Mg), manganese (Mn), molybdenum (Mo), vanadium (V), zinc (Zn)] and metalloids arsenic (As) were determined by inductively coupled plasma–mass spectrometry (ICP OES) after microwave digestion. Moreover, we assessed the associated risk regarding the elemental intake of these elements through the smoke, using the hazard quotient (HQ), hazard index (HI), Total Hazard Index (HIt), and carcinogenic risk (CR). All samples had results for HQ and HIt < 1, indicating a non-potential health risk. However, the carcinogenic risks posed by As and Cr via the three exposure pathways (except for inhalation exposure to children and adults, and by Cr via ingestion and inhalation for children and adults) exceeded the standard threshold. In conclusion, continuous exposure of firefighters or children to smoke from fires containing high concentrations of heavy metals such as As and Cr can be harmful to health. The study used animal tissues; thus, new methods must be developed to quantify the concentration of heavy metals deposited in human tissue when humans are exposed to smoke from fires.

## 1. Introduction

In the last decade, the wildfires incidence in Brazil increased badly, and since 2019, with unprecedented magnitude in the Amazon and Pantanal [1]. In addition to the consequences of forest fires involving burned areas and dead animals, there is the pollution resulting from smoke that has a direct impact on the health of the population and especially the firefighters who work daily fighting fires. In fact, exposure to fumes causes increased mortality, hospital admissions, and emergency room visits due to respiratory and cardiovascular diseases, as well as reduced lung function [2].

Exposure to carbon monoxide (CO), ammonia (NH_3_), carbon dioxide (CO_2_), and hydrogen sulfide (H_2_S), as well as respirable particulate matter (RPM), nitrogen oxides (NO_x_), polycyclic aromatic hydrocarbons, benzene, and acrolein, can cause difficulties breathing, exhaustion, dizziness, and other flu-like symptoms. In addition, there is a risk of exposure to certain heavy metals and metalloids, such as cadmium (Cd), lead (Pb), cobalt (Co), chromium (Cr), molybdenum (Mo), nickel (Ni), and arsenic (As), among other elements [3,4].

The occupational risk to which firefighters are exposed daily has been known for a long time—stress, fatigue, emotional and physical injuries; however, due to smoke emissions, this category of professionals also has numerous cases of chronic diseases, cancer, increased cardiopulmonary morbidity, and mortality [5,6,7].

This is due to the fact that wildfire, structure, and vehicle fires, as well as other fires, produce smoke from the incomplete combustion of different items, and it is agreed that this smoke contains a variety of compounds that are unsafe for humans [8,9]. According to the International Agency for Research on Cancer (IARC) Monographs program, this mix of substances can include many agents already classified as known (Group 1), probable (Group 2A), or possible (Group 2B) carcinogens, including heavy metals [10,11].

The most recent IARC report of 1 July 2022 changed the firefighting classification of possible (Group 2B) to a carcinogen to humans (Group 1) [11], corroborating with several studies that observed a higher incidence of cancer and/or risk of mortality among firefighters [6,10,12]. However, despite the risks, the use of personal protective equipment in firefighting is often unfeasible or ignored, especially in cases of forest fires [9]. This is an extremely important aggravating factor, since firefighters are exposed to metals and other hazardous products not only through inhalation but also predominantly through dermal contact, in addition to ingestion and contact with contaminated surfaces [9,11], which are worsened because of the lack of protective equipment.

According to Fangchao et al.’s (2006) [13] study on cancer incidence among Florida female firefighters, the firefighters had a significantly increased risk of cancer overall, as well as Hodgkin’s lymphoma disease and thyroid and cervical cancers, compared with the general population. In fact, firefighters are susceptible to hazardous constituents via the inhalation of various levels of particulates containing metals. Almost 70% of smoke particulates released during a wildfire are considered ultrafine particles, small enough to be inhaled into the lungs and translocate to practically all organs [14]. These ultrafine particles are the most likely cause of metal fume fever, a disease of lung inflammation, and although exposure is commonly attributed to welding, studies also showed that biomass combustion emitted smaller particles that have the greatest risk of inhalation [15], thus requiring more studies to assist in understanding of the magnitude of the problem.

Wood-smoke extract and derived components as particulate matter, when analyzed in in vitro experiments using human cell lines, demonstrated the ability to induce oxidative stress [16] and deoxyribonucleic acid (DNA) damage [17,18]. In the meantime, there are no experimental models involving the quantification of concentrations of heavy metals absorbed by dermal contact, inhalation, and the digestive tract after exposure to wood and charcoal smoke, and there is a lack of studies that have evaluated the risks considering the types of wood or materials to which the firefighters are exposed during the burning of these materials.

Finally, during a fire occurrence, physical activity increases ventilation, which leads to a proportional increase in the amount of pollutants inhaled [19]. In fact, according to Barros et al. (2021) [9], firefighters are exposed to heavy metals from various routes, such as inhalation, dermal, and ingestion, which may be posing firefighters to risk of inhaling air pollutants [11]. In addition, compared to adults, the implications of heavy metals in regard to children’s health are more severe [20]. The element consequences on children’s health include many illnesses, such as neurocognitive disorders, mental retardation, respiratory problems, cancer, cardio-metabolic risks, and diabetes [21]. In fact, according to Centers for Disease Control and Prevention (CDC), extra care must be taken to protect children against wildfire smoke [22]. Children with asthma, allergies, or chronic health issues may have more trouble breathing when smoke or ash is present [22,23].

To date, there are no experimental models that have studied the value of the concentration of heavy metals that can be deposited in human tissue through inhalation, ingestion, and dermal systems when exposed to smoke from wood and charcoal combustion. In addition, there are no risk calculations for human health due to ingestion of, inhalation of, and dermal contact with heavy metals from smoke from forest fires. However, to verify the concentration of heavy metals that can be adhered to biological tissues when exposed to smoke, animal tissues can be used to simulate human tissues due to their similarity. In fact, the tissues animal equivalent can be viewed as physiologically comparable to the natural human [24,25,26,27] and therefore is a suitable alternative for risk testing.

In view of the above, this work aimed to (i) quantify heavy metals in beef and swine meat exposed to smoke from some Brazilian Cerrado woods and also wood treated with chrome copper arsenate; (ii) obtain the value of the concentration of metals that come from smoke acquired from the subtraction of fresh meat (raw) and meat roasted in the presence of smoke; and (iii) investigate the potential health risk caused by the ingestion and dermal and inhalation contact of heavy metals from the combustion of wood, considering as a population the Fire Department professionals and children. As described above, firefighters are the most exposed daily to fumes from various materials.

## 2. Materials and Methods

### 2.1. Justification of the Choice of Biological Tissue Samples

There is little information in the literature on in vitro studies, experimental models, animals and humans that consider the ingestion, and the inhalation and dermal absorption of metals through wood or charcoal smoke. Given the above, according to Scalia et al. (2015) [26], swine and bovine tissue have molecular similarities with that of humans [24,25,26,27]. Therefore, cuts of beef topside and pork loin were selected so that the difference in concentration by deposition of metals present in the fine particulate material could be evaluated, simulating a fire-exposure scenario.

### 2.2. Sample Acquisition and Preparation

Three samples of beef topside and pork loin were purchased from different butcheries of Campo Grande, MS, in the Midwest region of Brazil, while the following woods were acquired through direct purchase in commercial establishments in Campo Grande, Brazil: *Eucalyptus citriodora* wood, *Guazuma ulmifolia* wood, *Anadenanthera falcata* wood, and treated *Eucalyptus* (CCA-treated *Eucalyptus* wood) with Chromed Copper Arsenate (CCA). In addition, two charcoals were purchased: *Eucalyptus citriodora* charcoal and *Guazuma ulmifolia* charcoal.

Fresh and roasted meats were divided into groups as follows; the first being raw meats (beef topside and pork loin); the second group being beef topside and pork loin roasted with *Eucalyptus* citriodora wood; the third group being beef topside and pork loin roasted with *Guazuma ulmifolia* wood; the fourth group being beef topside and pork loin roasted with *Anadenanthera falcata* wood; the fifth group being beef topside and pork loin roasted with treated *Eucalyptus* (CCA-treated *Eucalyptus* wood); the sixth group being beef topside and pork loin roasted with *Eucalyptus citriodora* charcoal; and the seventh group being beef topside and pork loin roasted with *Guazuma ulmifolia* charcoal. For sample preparation, aliquots collection, roasted conditions, and dimensions of masonry barbecue, all the entire procedures were performed according to the methodology proposed by Leite et al. (2020) [28], where meat samples were placed on a stainless steel grid, at a height of 40 cm from wood or charcoal, at a temperature of 280–300 °C. For each experiment with each type of wood and meat, the barbecue was cooled, cleaned, and sanitized, removing any particulate matter, such as ash, debris, or wood or charcoal residues. After the procedure, both raw and roasted meats were sliced using a scalpel with stainless steel blades. Then they were weighed and homogenized (Thermomix TM5 equipment-Vorwerk L.L.C., Wuppertal, Germany) and stored in a universal plastic collector and frozen at −20 °C until analysis.

#### Metals and Metalloids Accumulation

As a hypothesis, it was considered that inhalation, absorption through dermal contact, and ingestion of metals by adults that are firefighters and children result from the smoke from the difference in metal concentration between what was quantified for metals in meats exposed to smoke from burning biomass and concentrations obtained in raw meats. Therefore, the following procedure was performed:

1. Quantify metals in pork loin and beef topside raw muscles.
2. Quantify metals in pork and beef, both roasted using wood *Eucalyptus citriodora*, *Guazuma ulmifolia*, *Anadenanthera falcata* and CCA-treated *Eucalyptus* and charcoal *Eucalyptus citriodora*, and *Guazuma ulmifolia*;
3. Subtraction of the quantification of heavy metals from raw meats and meats roasted with different types of wood and charcoal.

Therefore, the concentration of heavy metals in the smoke was obtained by the difference in the quantification of heavy metals in raw meats and roasted meats.

### 2.3. Microwave Digestion and Elemental Measurement by Using ICP OES

Procedures were taken as described by Leite et al. (2020) [28]. About 300 mg of each meat sample was accurately weighed in a Teflon digestion vessel. Next, 2 mL of HNO_3_ (65% Merck, Darmstadt, Germany), 1.5 mL of H_2_O_2_ (30% Merck, Darmstadt, Germany), and 2 mL of ultrapure deionized water (18 MΩcm, Milli-Q Millipore, Bedford, MA, USA) were added. Digestion of samples was carried out in a microwave digestion system. All the digestion analyses steps were conducted in triplicate. The procedures for quantifying Al, As, Cr, Cu, Fe, Mg, Mn, Mo, V, and Zn in wood smoke through particulate matter obtained from roasted meats, using ICP OES, analytical calibration curve, the limit of detection (LOD), limit of quantification (LOQ), and correlation coefficient (R^2^), are described by Leite et al. (2020) [28].

An addition/recovery procedure was carried out for the elements under study by spiking 0.5 mg/L and 1.0 mg/L of each analyte in the pork sample. The recovery interval of the spikes is presented in Table 1 below, which is between 80 and 120%, the previously established limit proposed by the Union of Pure and Applied Chemistry (IUPAC) and the Association of Official Analytical Chemists (AOAC) [29,30].

### 2.4. Human Health Risk Assessment

The health risk assessment due to inhalation, ingestion due to particle deposition, and dermal absorption of heavy metals from smoke resulting from burning different types of firewood and charcoal was performed for children (up to 15 years old) and adults [31,32,33]. Thus, the health risk assessment was estimated using the following equations: Equation (1)—chemical daily intake (CDIing); Equation (2)—dermal absorbed dose of trace elements in particles adhered to exposed skin (DADderm); and Equation (3)—inhalation of resuspended particles through mouth and nose (Dinh) [34,35]. The cutaneous exposure, inhalation, and ingestion of these airborne particles are common routes of occupational exposure [9,11,36], and firefighters are exposed to these three pathways; therefore, the dose received through each of the three paths was calculated using Equations (1)–(3), as follows:

(1) $$\mathrm{CDIing}=\frac{C\times \mathrm{IngR}\times \mathrm{EF}\times \mathrm{ED}}{\mathrm{BW}\times \mathrm{AT}}\times \mathrm{CF}$$

(2) $$\mathrm{DADderm}\;=\frac{C\times \mathrm{SA}\times \mathrm{AF}\;\times \mathrm{ABS}\times \mathrm{EF}\times \mathrm{ED}}{\mathrm{BW}\times \mathrm{AT}}\times \mathrm{CF}$$

(3) $$\mathrm{Dinh}\;=C\times \frac{\mathrm{InhR}\times \mathrm{EF}\times \mathrm{ED}}{\mathrm{PEF}\times \mathrm{BW}\times \mathrm{AT}}$$

where C is the average concentration of metal in samples of raw or roasted meat with different types of wood and charcoal (mg/kg) quantified by ICP OES. BW is the body weight (15 kg for children and 70 kg for adults), AT is the averaging time (for non-carcinogenic risks, AT = ED × 365 days; and for carcinogenic risks, AT = 70 × 365 = 25,550 days), and CF is a scaling factor (10^−6^ kg mg^−1^). To characterize time and duration of exposure doses, in this study, we established EF as the exposure frequency (120 days year^−1^) and ED as the exposure duration (6 years for children and 24 years for adults). Here, the 120 days was considered, as it corresponds to the period of days that remains the dry season in which the highest number of fires occurs in Brazil, that is, between the months of July and October [34,37,38]. Therefore, the results can be considered “conservative estimates” due to lower than annual EF [39]. In Equation (1), IngR is the ingestion rate, which was fixed to 200 mg day^−1^ for children and 100 mg day^−1^ for adults [32]. In Equation (2), SA corresponds to the skin-surface area in contact with dust (5700 cm^2^ for adults and 2800 cm^2^ for children) [34], AF to the skin adherence factor (0.2 mg cm^−2^ day^−1^), and ABS to the dermal absorption factor (0.03 for As, 0.001 for Cd, and 0.01 for the rest of the elements) [34]. In the Dinh calculation Equation (3), the EF of 180 day year^−1^ was adapted from the method described by Zheng et al. [35]. In addition, in this equation, the PEF, i.e., particle emission factor, was 1.36 × 10^9^ m^3^ kg^−1^ [40], and the InhR, i.e., inhalation rate, was 7.6 m^3^ day^−1^ for children and 20 m^3^ day^−1^ for adults [32].

The hazard quotient (HQ) level due to each of the contamination routes was calculated using Equation (4) for non-carcinogenic damage in humans:

(4) HQing=CDIing/RfDoHQderm=DADderm/(RfDo×GIABS)HQinh=Dinh/RfCi

where RfCi is the reference concentration for inhalation in (mg m^3^), RfDo is the oral reference dose in (mg kg^−1^ day^−1^), and GIABS is the gastrointestinal absorption factor. They were all established by the United States Environmental Protection Agency (USEPA) in the “Regional Screening Levels (RSLs)—Summary Table”, updated version, in May 2022 [41].

To assess the overall potential for non-carcinogenic risks posed by an individual simultaneously exposed to two or more elements, the hazard index (HI) was the sum of HQs for multi-element exposure. However, to obtain a whole Total Hazard Index (HIt) that was able to estimate aggregate exposure pathways, the HIt was estimated as the sum of HQs (ingestion, dermal, and inhalation), also assuming additive effects [31,42]. If the value of the HI or HQ is greater than 1, it indicates potential non-carcinogenic damage on human health, whereas HQ < 1 does not indicate risk [42,43].

Since the International Agency for Research on Cancer (IARC) lists inorganic arsenic, cadmium, chromium (VI), nickel, and lead as human carcinogens [8], it is possible to estimate the carcinogenic risk (CR), which is the probability of an individual developing cancer during his or her lifetime due to exposure to a chemical known to be carcinogenic. The CR was calculated considering inhalation, ingestion, and dermal absorption through daily exposure over the years of life for children and adults, using Equation (5):

(5) CRing=CDIing × SFo CRderm=DADderm×(SFo/GIABS)CRinh=Dinh × SF

where the slope factor (SF) (mg/kg/day) was used for arsenic and the other three heavy metals considered carcinogenic elements or probably carcinogenic to humans, and their available SF values are the following: As = 1.5 mg/kg/day, Cd = 6.1 mg/kg/day, Cr = 0.5 mg/kg/day, and Pb = 0.0085 mg/kg/day [41,44].

According to the USEPA [45], a CR value between 10^−6^ and 10^−4^ indicates that the cancer risk is within a tolerable range. However, when the estimated values of CR are greater than 10^−4^, this shows that human tolerance is exceeded.

### 2.5. Statistical Analysis

The data were analyzed by one-way ANOVA, using the GraphPad Prism 8 software version 8.0.1 (GraphPad Software, San Diego, CA, USA), to compare average values with a post hoc Tukey test for parametrical distribution. A significant difference was determined when the *p*-value was inferior to 0.05.

## 3. Results

### 3.1. Elemental Content from Smoke from Burning Wood/Coal in a Controlled Environment

Table 2 shows the concentrations of metals (Al, Cr, Cu, Fe, Mg, Mn, Mo, V, and Zn), and metalloid (As) quantified in samples of beef topside and pork loin fresh muscles exposed to Cerrado firewood smoke, firewood for domestic use, treated wood for structural use, and charcoal. The concentration levels of Cd, Co, Ni, and Pb in all samples were below the limit of detection.

In Table 2, the metal contents in *Eucalyptus citriodora* wood smoke from the beef top-side samples were found in the following decreasing order: Mg > Zn > Fe > Al > Cu > Mn > Mo > As > V. This was different from the content quantified in pork loin samples, with Mg > Zn > Fe > Al > Mn > V > As > Cu > Cr. The smoke of *Guazuma ulmifolia* wood in beef topside samples presented elemental concentrations as follows: Mg > Zn > Al > Cu > Mo > Fe > V > Mn > As. Meanwhile, the elements in the smoke from pork loin decrease in the following order: Mg > Zn > Al > Fe > V > As > Mn > Cr > Cu. The metal content decreased in the order of Mg > Al > Zn > Fe > Cu > Mo > Mn = V > As for wood smoke from *Anadenanthera falcata* in beef topside, but in the smoke from pork loin sample, the elements were quantified as follows: Mg > Al > Fe > Zn > V > As > Cr > Cu. Regarding *Eucalyptus* smoke treated with CCA, the following descending order of elements was obtained for beef topside: Mg > Zn > Fe > As > Al > Cu > Cr > V > Mn. Meanwhile, for pork loin, the order was Mg > As > Al > Zn > Fe > Cr > Cu > V.

Considering the charcoal group, the concentration of metal in smoke from *Eucalyptus Citriodora* detected in beef topside and pork loin decreases for both as follows: Mg > Zn > Fe > Al > As > Mn > Cu > V > Cr; and Mg > Al > Zn > Fe > As > Mn > V > Cr > Cu. Furthermore, the metals quantified in the smoke of charcoal samples from the Cerrado *Guazuma ulmifolia* decreased in following order for beef topside: Mg > Zn > Fe > Al > Cu > V > Cr > Mn > As. Meanwhile, for pork loin, the order was Mg > Al > Fe > Zn > As > V > Cu > Mn > Cr.

The Al content in smoke deposited in beef topside ranged from 2.629 ± 0.053 to 32.670 ± 0.611 mg/kg, and for pork loin from 2.435 ± 0.132 to 47.440 ± 0.716 mg/kg. The one-way ANOVA showed that the biomass type played a role in the Al content. The fuel that was responsible for the major variation in beef topside and pork loin samples, respectively, was a smoke from the Cerrado charcoal known as *Guazuma ulmifolia* (32.670 ± 0.611 mg/kg–47.440 ± 0.716 mg/kg, *p* < 0.0001), followed by the *Anadenanthera falcata* smoke, another typical wood from Brazilian Cerrado (16.637 ± 0.226 mg/kg in beef topside, and 14.682 ± 0.162 mg/kg in pork loin, *p* < 0.0001).

As shown in Table 2, the difference in the As concentration from smoke in beef topside samples rose significantly with CCA-treated *Eucalyptus* (21.163 ± 0.252 mg/kg) and *Eucalyptus citriodora* charcoal (1.359 ± 0.033 mg/kg) (*p* < 0.0001). Meanwhile, the concentration difference measured in pork loin samples also showed higher As concentrations with smoke from CCA-treated *Eucalyptus* (16.524 ± 0.155 mg/kg), followed by the smoke of charcoals of *Eucalyptus citriodora* and *Guazuma ulmifolia* (1.512 ± 0.067–1.629 ± 0.048 mg/kg), respectively (*p* < 0.0001).

The smoke concentration of Cr in beef topside samples ranged from 0.033 ± 0.006 to 1.092 ± 0.026 mg/kg, whereas the smoke from firewood samples did not have a detectable amount of this element, except for treated *Eucalyptus* wood. For pork loin samples, the Cr content in smoke ranged similarly, from 0.084 ± 0.004 to 1.042 ± 0.087 mg/kg. Statistically, the Cr content quantified in smoke only differs for treated *Eucalyptus* (*p* < 0.0001) in both types of meat.

The results for the Cu contents detected in smoke for beef topside samples were higher in *Eucalyptus citriodora* wood 2.291 ± 0.020 mg/kg, followed by treated *Eucalyptus* 1.860 ± 0.053 mg/kg, whereas smoke from swine samples has higher amounts of Cu influenced by CCA-treated *Eucalyptus*, 0.943 ± 0.013 mg/kg, and *Guazuma ulmifolia* charcoal, 0.508 ± 0.088 mg/kg.

The minimum Fe level in meat samples was 0.905 ± 0.159 mg/kg for smoke from *Guazuma ulmifolia* wood in beef topside and 2.841 ± 0.191 mg/kg for the smoke from *Eucalyptus citriodora* wood deposited in pork loin, while the maximum level in smoke was for *Guazuma ulmifolia* charcoal, with 53.111 ± 0.254 mg/kg and 9.801 ± 0.382 mg/kg in beef topside and pork loin, respectively (Table 2).

The Mg content was high in smoke from all samples; however, the biomass that most influenced the Mg values was *Guazuma ulmifolia* charcoal (184.385 ± 6.549 mg/kg) and *Eucalyptus citriodora* wood (163.701 ± 3.350 mg/kg; *p* < 0.0001), both deposited in beef topside, followed by *Guazuma ulmifolia* charcoal smoke in pork loin (172.659 ± 10.835 mg/kg; *p* < 0.0001).

The Mn contents found in the smoke are shown in Table 2. The major Mn concentration for beef topside and pork loin samples occurred in the smoke of all *Eucalyptus citriodora* samples. The Mn for the smoke of *Eucalyptus citriodora* wood in beef topside was 1.275 ± 0.004, and for pork loin, it was 0.803 ± 0.005 mg/kg, while the *Eucalyptus citriodora* charcoal was 1.050 ± 0.011 mg/kg in beef topside and 1.051 ± 0.008 mg/kg in pork loin.

Mo values were detected in the smoke from beef topside samples only. The Mo concentration was more influenced by the group of woods, presenting maximum values of 1.206 ± 0.006, 0.934 ± 0.009, and 0.904 ± 0.018 mg/kg for *Eucalyptus citriodora*, *Guazuma ulmifolia*, and *Anadenanthera falcata*, respectively.

The V present in the smoke came mainly from *Guazuma ulmifolia* charcoal; for beef topside, it was 1.023 ± 0.38 mg/kg, and pork loin, it was 1.421 ± 0.022 mg/kg, *p* < 0.0001. Likewise, in the bottom half of Table 2, for Zn, the smoke in beef topside showed higher concentrations of Zn for *Guazuma ulmifolia* charcoal, with 81.272 ± 0.086 mg/kg; CCA-treated *Eucalyptus*, with 55.275 ± 0.387 mg/kg; and *Eucalyptus citriodora* charcoal, with 54.243 ± 0.416 mg/kg. For pork loin, the highest Zn concentrations were for *Guazuma ulmifolia* charcoal, with 9.699 ± 0.357 mg/kg, and wood, with 9.786 ± 0.459 mg/kg.

### 3.2. Human Health Risk Assessment

Table 3 and Table 4 show the hazard quotient (HQ) results for children and adults exposed to smoke by ingestion, dermal exposure, and inhalation, while Table 5 shows the hazard index (HI) values for children and adults due to various exposures. These results, as described in Table 3 and Table 4, do not raise concerns, not showing non-carcinogenic risk by adding up the risk quotients for simultaneous exposure to multi-metals and different smoke exposure pathways.

The data presented in Table 6 demonstrate the carcinogenic risk (CR) results for As and Cr by smoke exposure from five Brazilian biomass fuels for children and adults.

## 4. Discussion

### 4.1. Elemental Content from Smoke from Burning Wood/Coal in a Controlled Environment

The results above show differences in the element concentrations found in smoke between the two types of muscles (beef topside and pork loin) due to the leaching of minerals in the broth, as well as the influence of the different chemical compositions of the species, such as the percentage of fat lost during heating [46], along with the influence of the heat processing method, as even variations in time, temperature, oxygenation, humidity, the direction of the smoke during the experiment, and the combustion phases for each fuel have an impact [47]. For this reason, the values of each element cannot be compared between different animal species, but only between biomass types for each sample. The physical properties of fresh meat, such as water, water activity, water-holding capacity, meat texture, moisture, firmness, pH, fibers, etc., can influence the final values of the elements [48,49]. Therefore, these differences between the concentrations of macro- and microelements between the two types of meat can be explained according to their physical properties, such as the different amounts of water in fresh meat, among others previously mentioned.

Among the carcinogens most important to air toxicity regulatory programs are metals (e.g., As, Cd, Cr, Be, and Ni) [50,51]. In addition to that, dermal exposure, inhalation, and ingestion of these aerial particulate matters are common routes of occupational exposure [9,11,36].

For this reason, some studies among firefighters have found increased levels of metals after fire occurrence. A study conducted in New Mexico evaluated the association of urinary concentrations of metals with exposure to smoke. For comparison purposes, the results they obtained were classified based on data from the Third National Health and Nutrition Examination Survey (NHANES III), where values above the 95th percentile of population levels found from NHANES III were classified as “above reference”. That way, concentrations higher than expected were observed for nickel, cesium, chromium, and uranium in both firefighters and non-firefighters at the burned area. Meanwhile, As and Cd levels were significantly related to smoke exposure for National Guard members, just as Cs and As were related to fire smoke exposure for firefighters [52].

The indirect method to determine the concentration of heavy metals in smoke from burning wood/coal in a controlled environment makes it clear that elements such as Al, As, Cr, Cu, Fe, Mg, Mn, Mo, V, and Zn are present in the smoke, corroborating Wolf’s study, which quantified elements such as Mo, As, Ni, Hg, Cs, Ba, Cd, Cr, Co, Pb, U, W, Be, Sb, Tl, and Pt in the urine of firefighters. That is, such elements can be inhaled, ingested, or absorbed dermally. Also according to Wolf et al. (2004) [52], the urine heavy metal concentrations in decreasing order of elements was Mo > As > Ni > Hg > Cs > Ba > Cd > Cr > Co > Pb > U > W > Be > Sb > Tl > Pt for the general population and Mo > As > Hg > Ni > Cs > Ba > U > Pb > Cr > Cd > Co > W > Be > Sb > Tl > Pt for firefighters, thus differing from what was found in our study.

Although Wolf et al. (2004) [52] emphasize that there was no acute harm to the individuals, as they did not need immediate medical follow-up, we believe that it is dangerous to consider that the metals present in the urine of firefighters have little clinical and/or public health importance, since the health damage suffered by firefighters during their years of service is known and measurable, as mentioned by Dobraca et al. (2015) [53] in a Firefighter Occupational Exposures study; higher blood concentrations of Cd, Pb, and Hg were seen in firefighters aged 50 years or older, indicating cumulative effects and thus contradicting the little clinical and/or public health importance that was stated by Wolf et al. (2004) [52]. Another significant result was found was for Cd, which showed higher blood values in firefighters who washed their hands less frequently during an occurrence [53], thus reaffirming the conclusion of previous studies on the risks associated not only with exposure to particulate matter through inhalation but also by dermal contact [54].

According to Burton et al. (2016) [55], large-scale wildfires seem to release elevated levels of metal(loid)s—which can be inhaled—such as in the Camp Fire, where researchers found in the air the heavy metals Mn and Ca even after the fire. Pb values were 50 times above the normal average near the area, and Zn increased at 150 miles away [56]. Another major concern involving occupational exposure is about the physical activity demanded during a fire incident, which increases ventilation, leading to the enlargement of the amount of pollutants inhaled, proportionately [19]. In the same way, children exposed to these areas raise concern, and although our study was not able to prove the risk of exposure for this group, studies are unanimous in stating that children are more susceptible to the risk of air pollution [57,58], including inhaling smoke from forest fires, as they are exposed more to outdoor environments, and, at the same time, they engage in more vigorous activities, thus inhaling more smoke and its constituents, being an aggravating factor the amount of pollutants inhaled per kilogram of body weight and the fact that all these substances can affect the developing lungs [57].

Intoxication by dermal contact was evidenced in the study by Fabian et al. (2010) [54], who evaluated the deposition of soot on protective equipment used by firefighters, as well as on the hands and gloves, making it possible to perceive that, in addition to the deposition of soot on the surfaces of the suits, there was still contamination through skin contact when removing these protective pieces of equipment. Moreover, considering this exposure pathway, according to the South African work that showed the influence of dermal exposure to mining metals on carcinogenic and non-carcinogenic risk, the dermal influence was greater than inhalation for both risks, both for children and for adults [59]. This highlights the need for further study of these exposure pathways, as well as constant monitoring and comparison with recommended maximum allowable limits for airborne metals, dust, and ash. Mainly during and after large fires, there is a need to protect firefighters and the residents of affected areas, especially children and professionals on duty, from heavy metal pollution in the environment.

Ingestion is a less quantifiable route of exposure during a fire; however, when fire fine particulates are inhaled, they may be carried through mucus and saliva into the digestive system and, in this way, absorbed into the body [60]. Also in the post-fire period, biomass combustion volatilizes the nutrients, releasing some metals into the atmosphere [61], causing these metals to become more mobile in recently burned areas, and they may be, in part, ingested. Furthermore, in association with ash dispersal and this increased overland contamination, there is a risk of bioaccumulating in the food web, possibly causing water quality issues and possibly contributing to human and environmental health concerns [62]. Some of these chemicals cause deleterious effects, and chronic human exposure, even at low doses, can be associated with chronic diseases and cancer [36,61]. In daily life, humans are exposed to heavy metals and their mixtures in air, soil, water, and food, and the consumption of contaminated products/things can result in health issues such as diabetes, cardiovascular, neuronal, and renal disorders, in addition to risk of cancer [63].

Although our study did not demonstrate risk from exposure to metals present in particulate matter from forest fires and structural fires, considering acute and chronic exposure, as reviewed in many previous works [3,4,14,19,52,54,55], it is possible to see that firefighters’ occupational exposure is capable of increasing metal concentrations in urine and blood, having cumulative effects. Some heavy metals, such as Pb, Cr, and Cd, are considered toxic by numerous routes of exposure, including ingestion, inhalation, and skin absorption, with more severe effects on children’s health. The harmful effects of the elements on children’s health include mental retardation, neurocognitive disorders, behavioral disorders, respiratory problems, cancer, and cardiovascular disease, due to their high toxicity potential, widespread use, and high prevalence [20].

Therefore, we would like to point out that there is a need for more research that tests metals during and after the fire for a better evaluation of the toxicity in each condition. Finally, this study’s limitations included mostly our lack of data capable to scale all the particles emitted during the fuel burning process; this lack may have led to an underestimation of the elemental concentration and also the associated risk. For this reason, epidemiological studies were reviewed to help understand the incidence of the health problems associated with firefighters. Second, the findings and their implications should be discussed in the broadest context possible, from the perspective of previous studies and of the working hypotheses. To continue clarifying the theme, future research may also be needed.

### 4.2. Health Hazards Considering Elemental Intake through Inhalation, Ingestion, and Dermal Exposure

According to Table 3 and Table 4, the hazard quotient (HQ) values of the metals in all the pathways are below 1; therefore, they represent acceptable levels (no concern). Similar to Table 5, all values for HI and Hit are < 1; thus, the non-carcinogenic adverse effect due to the exposures pathways or chemicals is assumed to be negligible. Therefore, even if an individual is simultaneously exposed to the smoke of fires containing multi-elements by two or more pathways, there is no evidence of potential non-carcinogenic damage to human health.

The carcinogenic risk in Table 6, considering metal in smoke from wood and charcoal that was deposited in beef as a model of metal deposition, showed that the ingestion of smoke containing As by adults is beyond the established tolerable values for a single element (1 × 10^−6^) [64], while the CR values for chromium are below these values for adults and children. The same was observed for smoke deposition in pork per ingestion. The risk of dermal exposure to As for adults is beyond the established tolerable values for wood and charcoal in both models of metal deposition (beef and pork). However, the highest carcinogenic risk value for adults when considering dermal contact (charcoal, *Eucalyptus citriodora*, CR = 1.21 × 10^−3^, in beef) exceeds the tolerable values. Continuing the risk assessment for dermal exposure of adults and children to chromium with beef and pork samples roasted with treated wood and beef roasted with *Guazuma ulmifolia* charcoal, they all raise concern, as they are considered higher than the maximum limit allowed of 10^−6^ for a single element [64]. Regarding the carcinogenic risk due to inhalation of As and Cr in smoke, all values are less than 10^−6^, with no health risk.

According to the carcinogenic risk classification proposed by Rapant et al. (2010) [65], it is possible to observe that CR showed a different distribution among smoke samples, which are considered of very high risk (>10^−3^) for 1 sample, medium risk (10^−5^ to 10^−4^) for 20 samples, and low risk (10^−6^ to 10^−5^) for 12 samples, with the remainder being considered very low risk (<10^−6^). Our results are in accordance with the studies reviewed by us, and the recent change in the IARC risk classification for firefighters supports the hypothesis that wildfires may be more dangerous than we imagined [10,11].

Although the risks of exposure to smoke are established [10,66,67], the increase in the number of great proportions of wildfires presents new faces of the problem (consequences) that we were not aware of until then. The last California Air Resources Board report, which analyzed substances from the 2018 Camp Fire wildfire, found dangerous levels of heavy metals, mainly lead, in the air [56]. Another recent study noted that there is a difference between smoke from forest fires compared to smoke from other sources and that the damage from exposure to particulate matter from wildfires is greater for this type of smoke [68,69].

Furthermore, our results are in accordance with our literature review, which showed that metal concentration in fires can vary for many reasons, and it is a red flag that requires attention. So, the evidence presented thus far supports the fact that firefighters are in occupational danger. Even though our results are limited to wildfires in the Cerrado, Central–West Region of Brazil, and structural/building burns containing wood chemically treated and Cerrado’s traditional wood, we were able to detect the presence of Al, As, Cr, Cu, Fe, Mg, Mn, Mo, V, and Zn in the smoke from fires. In spite of its limitations, the study certainly adds to our understanding of metal smoke exposure, its characteristics, and it variables.

## 5. Conclusions

The study showed that, in the smoke produced by different types of wood and charcoal, there are elements such as Al, Cr, Cu, Fe, Mg, Mn, Mo, V, and Zn that can be deposited in the dermis or be ingested and inhaled by adults and children.

The health risk assessment for all the samples (beef and pork), looking at the three pathways, indicated that there is no dangerous single heavy metal, but their carcinogenic risk, as indicated by the calculations, calls for concern. In fact, there are no safe limits for the ingestion, inhalation, and even dermal contact of heavy metals when exposure is continuous and for long periods.

Moreover, further effort should be aimed at measuring peak smoke exposure for all types of fires—we believe that our measurements have not identified the upper range of heavy metals that firefighters come into contact with through smoke exposures. Our data showed concentrations above to the risk limit, considering only the material deposited in meats; therefore, future smoke-exposure characterization work should consider ways to measure this exposure more completely in order to identify and quantify these chemicals for a better assessment of its impact on human health. This effort becomes more urgent when we think about the gap in exposure assessment methodologies aimed at children and the particularity of risks of exposure to the child population, which is severely hit.

## Figures and Tables

**Table 1:** Validation of methods using the spike concentrations of analytes.

Analyte	Spike Recovery (%)	
	0.5 mg/L	1.0 mg/L
Al	108	108
As	102	103
Cd	91	93
Co	99	99
Cr	107	108
Cu	104	104
Fe	106	105
Mg	101	99
Mn	100	101
Mo	111	112
Ni	101	101
Pb	90	93
V	110	110
Zn	102	99

**Table 2:** Concentrations of chemical elements obtained from the difference between “roasted” animal tissue minus fresh raw tissue.

	Concentrations (Average mg/kg ± SD)						***p-*** **Value**
	Wood				Charcoal		
	*Eucalyptus citriodora*	*Guazuma ulmifolia*	*Anadenanthera falcata*	CCA-Treated Eucalyptus	*Eucalyptus citriodora*	*Guazuma ulmifolia*	
Al							
Beef	2.629 ± 0.053 ^a^	6.711 ± 0.061 ^b^	16.637 ± 0.226 ^d^	11.084 ± 0.317 ^c^	6.876 ± 0.085 ^b^	32.670 ± 0.611 ^e^	<0.01 ^1^
Pork	2.435 ± 0.132 ^a^	8.364 ± 0.245 ^b^	14.682 ± 0.162 ^d^	11.944 ± 0.134 ^c^	8.302 ± 0.159 ^b^	47.440 ± 0.716 ^e^	<0.01 ^1^
As							
Beef	0.845 ± 0.024 ^c^	0.088 ± 0.017 ^a^	0.203 ± 0.008 ^a,b^	21.163 ± 0.252 ^e^	1.359 ± 0.033 ^d^	0.485 ± 0.048 ^b^	<0.01 ^1^
Pork	0.428 ± 0.017 ^a,b^	0.582 ± 0.024 ^b^	0.306 ± 0.046 ^a^	16.524 ± 0.155 ^d^	1.512 ± 0.067 ^c^	1.629 ± 0.048 ^c^	<0.01 ^1^
Cd							
Beef	<LOD	<LOD	<LOD	<LOD	<LOD	<LOD	
Pork	<LOD	<LOD	<LOD	<LOD	<LOD	<LOD	
Co							
Beef	<LOD	<LOD	<LOD	<LOD	<LOD	<LOD	
Pork	<LOD	<LOD	<LOD	<LOD	<LOD	<LOD	
Cr							
Beef	<LOD ^a^	<LOD ^a^	<LOD ^a^	1.092 ± 0.026 ^c^	0.033 ± 0.006 ^a^	0.767 ± 0.038 ^b^	<0.01 ^1^
Pork	0.084 ± 0.004 ^a^	0.248 ± 0.004 ^b^	0.255 ± 0.005 ^b^	1.042 ± 0.087 ^c^	0.262 ± 0.014 ^b^	0.386 ± 0.011 ^b^	<0.01 ^1^
Cu							
Beef	2.291 ± 0.020 ^e^	1.587 ± 0.024 ^c^	1.430 ± 0.017 ^b^	1.860 ± 0.053 ^d^	1.035 ± 0.025 ^a^	1.062 ± 0.036 ^a^	<0.01 ^1^
Pork	0.199 ± 0.009 ^a^	0.228 ± 0.022 ^a^	0.167 ± 0.012 ^a^	0.943 ± 0.013 ^c^	0.245 ± 0.029 ^a^	0.508 ± 0.088 ^b^	<0.01 ^1^
Fe							
Beef	5.688 ± 0.588 ^c^	0.905 ± 0.159 ^a^	3.376 ± 0.352 ^b^	36.846 ± 0.677 ^d^	49.397 ± 0.535 ^e^	53.111 ± 0.254 ^f^	<0.01 ^1^
Pork	2.841 ± 0.191 ^a^	6.698 ± 0.274 ^c^	6.990 ± 0.310 ^c^	4.848 ± 0.147 ^b^	4.292 ± 0.359 ^b^	9.801 ± 0.382 ^d^	<0.01 ^1^
Mg							
Beef	163.701 ± 3.350 ^d^	89.620 ± 4.365 ^a^	101.292 ± 4.619 ^a^	130.909 ± 4.492 ^b^	146.796 ± 4.537 ^c^	184.385 ± 6.549 ^e^	<0.01 ^1^
Pork	91.005 ± 9.025 ^a^	154.479 ± 13.662^b,c^	100.856 ± 14.277 ^a^	132.764 ± 5.060 ^b^	142.845 ± 8.152 ^b^	172.659 ± 10.835 ^c^	<0.01 ^1^
Mn							
Beef	1.275 ± 0.004 ^e^	0.244 ± 0.007 ^a^	0.487 ± 0.007 ^c^	0.351 ± 0.001 ^b^	1.050 ± 0.011 ^d^	0.513 ± 0.014 ^c^	<0.01 ^1^
Pork	0.803 ± 0.005 ^d^	0.283 ± 0.007 ^b^	<LOD ^a^	<LOD ^a^	1.051 ± 0.008 ^e^	0.389 ± 0.010 ^c^	<0.01 ^1^
Mo							
Beef	1.206 ± 0.006 ^d^	0.934 ± 0.009 ^c^	0.904 ± 0.018 ^b^	<LOD ^a^	<LOD ^a^	<LOD ^a^	<0.01 ^1^
Pork	<LOD	<LOD	<LOD	<LOD	<LOD	<LOD	
Ni							
Beef	<LOD	<LOD	<LOD	<LOD	<LOD	<LOD	
Pork	<LOD	<LOD	<LOD	<LOD	<LOD	<LOD	
Pb							
Beef	<LOD	<LOD	<LOD	<LOD	<LOD	<LOD	
Pork	<LOD	<LOD	<LOD	<LOD	<LOD	<LOD	
V							
Beef	0.774 ± 0.015 ^c^	0.433 ± 0.010 ^a^	0.490 ± 0.037 ^a^	0.676 ± 0.010 ^b^	0.691 ± 0.006 ^b^	1.023 ± 0.038 ^d^	<0.01 ^1^
Pork	0.466 ± 0.012 ^a^	0.790 ± 0.015 ^c^	0.498 ± 0.013 ^a^	0.679 ± 0.015 ^b^	0.673 ± 0.018 ^b^	1.421 ± 0.022 ^d^	<0.01 ^1^
Zn							
Beef	18.962 ± 0.484 ^c^	7.750 ± 0.616 ^a^	13.598 ± 0.235 ^b^	55.275 ± 0.387 ^d^	54.243 ± 0.416 ^d^	81.272 ± 0.086 ^e^	<0.01 ^1^
Pork	6.056 ± 0.348 ^b^	9.786 ± 0.459 ^d^	4.731 ± 0.378 ^a^	7.023 ± 0.218 ^b,c^	7.410 ± 0.308 ^c^	9.699 ± 0.357 ^d^	<0.01 ^1^

<LOD: analyte concentration below the limit of detection. CCA = copper chrome arsenate. ^1^ Different letters (a, b, c, d, e, f) superscript in the same line represent statistic difference amongst groups (*p* < 0.01) by one-way ANOVA followed by post hoc Tukey’s test. However, comparison can be made only between the different fuels for the concentration of one element and not between different elements’ concentrations.

**Table 3:** Hazard quotient (HQ) values for Al, As, Cr, Cu, Fe, Mn, Mo, V, and Zn in children exposed to smoke via ingestion, dermal contact, and inhalation.

HQ for Children	Meat Type	Smoke Samples	Elements								
			Al	As	Cr	Cu	Fe	Mn	Mo	V	Zn
Ingestion	Beef	Wood Eucalyptus citriodora	1.15 × 10^−5^	1.24 × 10^−2^	-	2.51 × 10^−4^	3.56 × 10^−5^	3.99 × 10^−5^	1.06 × 10^−3^	6.78 × 10^−4^	2.77 × 10^−4^
		Wood Guazuma ulmifolia	2.94 × 10^−5^	1.29 × 10^−3^	-	1.74 × 10^−4^	5.67 × 10^−6^	7.63 × 10^−6^	8.19 × 10^−4^	3.80 × 10^−4^	1.13 × 10^−4^
		Wood Anadenanthera falcata	7.29 × 10^−5^	2.97 × 10^−3^	-	1.57 × 10^−4^	2.11 × 10^−5^	1.52 × 10^−5^	7.93 × 10^−4^	4.30 × 10^−4^	1.99 × 10^−4^
		Wood CCA-Treated Eucalyptus	4.86 × 10^−5^	3.09 × 10^−1^	1.59 × 10^−3^	2.04 × 10^−4^	2.31 × 10^−4^	1.10 × 10^−5^	-	5.93 × 10^−4^	8.08 × 10^−4^
		Charcoal Eucalyptus citriodora	3.01 × 10^−5^	1.99 × 10^−2^	4.85 × 10^−5^	1.13 × 10^−4^	3.09 × 10^−4^	3.29 × 10^−5^	-	6.06 × 10^−4^	7.93 × 10^−4^
		Charcoal Guazuma ulmifolia	1.43 × 10^−4^	7.08 × 10^−3^	1.12 × 10^−3^	1.16 × 10^−4^	3.33 × 10^−4^	1.61 × 10^−5^	-	8.97 × 10^−4^	1.19 × 10^−3^
Dermal	Beef	Wood Eucalyptus citriodora	3.23 × 10^−6^	1.04 × 10^−3^	-	7.03 × 10^−5^	9.97 × 10^−6^	2.79 × 10^−4^	2.96 × 10^−4^	7.31 × 10^−3^	7.76 × 10^−5^
		Wood Guazuma ulmifolia	8.24 × 10^−6^	1.08 × 10^−4^	-	4.87 × 10^−5^	1.59 × 10^−6^	5.34 × 10^−5^	2.29 × 10^−4^	4.09 × 10^−3^	3.17 × 10^−5^
		Wood Anadenanthera falcata	2.04 × 10^−5^	2.50 × 10^−4^	-	4.39 × 10^−5^	5.92 × 10^−6^	1.07 × 10^−4^	2.22 × 10^−4^	4.63 × 10^−3^	5.56 × 10^−5^
		Wood CCA-Treated Eucalyptus	1.36 × 10^−5^	2.60 × 10^−2^	1.79 × 10^−2^	5.71 × 10^−5^	6.46 × 10^−5^	7.70 × 10^−5^	-	6.39 × 10^−3^	2.26 × 10^−4^
		Charcoal Eucalyptus citriodora	8.44 × 10^−6^	1.67 × 10^−3^	5.43 × 10^−4^	3.18 × 10^−5^	8.66 × 10^−5^	2.30 × 10^−4^	-	6.53 × 10^−3^	2.22 × 10^−4^
		Charcoal Guazuma ulmifolia	4.01 × 10^−5^	5.95 × 10^−4^	1.25 × 10^−2^	3.26 × 10^−5^	9.31 × 10^−5^	1.13 × 10^−4^	-	9.66 × 10^−3^	3.33 × 10^−4^
Inhalation	Beef	Wood Eucalyptus citriodora	9.66 × 10^−8^	1.04 × 10^−5^	-	-	-	4.69 × 10^−6^	1.11 × 10^−7^	1.42 × 10^−6^	-
		Wood Guazuma ulmifolia	2.47 × 10^−7^	1.08 × 10^−6^	-	-	-	8.95 × 10^−7^	8.58 × 10^−8^	7.95 × 10^−7^	-
		Wood Anadenanthera falcata	6.11 × 10^−7^	2.49 × 10^−6^	-	-	-	1.79 × 10^−6^	8.31 × 10^−8^	9.01 × 10^−7^	-
		Wood CCA-Treated Eucalyptus	4.07 × 10^−7^	2.59 × 10^−4^	2.01 × 10^−6^	-	-	1.29 × 10^−6^	-	1.24 × 10^−6^	-
		Charcoal Eucalyptus citriodora	2.53 × 10^−7^	1.66 × 10^−5^	6.10 × 10^−8^	-	-	3.86 × 10^−6^	-	1.27 × 10^−6^	-
		Charcoal Guazuma ulmifolia	1.20 × 10^−6^	5.93 × 10^−6^	1.41 × 10^−6^	-	-	1.89 × 10^−6^	-	1.88 × 10^−6^	-
Ingestion	Pork	Wood Eucalyptus citriodora	1.07 × 10^−5^	6.26 × 10^−3^	-	2.19 × 10^−5^	1.78 × 10^−5^	2.52 × 10^−5^	-	4.09 × 10^−4^	8.85 × 10^−5^
		Wood Guazuma ulmifolia	3.67 × 10^−5^	8.50 × 10^−3^	3.63 × 10^−4^	2.50 × 10^−5^	4.19 × 10^−5^	8.85 × 10^−6^	-	6.93 × 10^−4^	1.43 × 10^−4^
		Wood Anadenanthera falcata	6.44 × 10^−5^	4.47 × 10^−3^	3.72 × 10^−4^	1.83 × 10^−5^	4.38 × 10^−5^	-	-	4.36 × 10^−4^	6.91 × 10^−5^
		Wood CCA-Treated Eucalyptus	5.24 × 10^−5^	2.41 × 10^−1^	1.52 × 10^−3^	1.03 × 10^−4^	3.04 × 10^−5^	-	-	5.95 × 10^−4^	1.03 × 10^−4^
		Charcoal Eucalyptus citriodora	3.64 × 10^−5^	2.21 × 10^−2^	3.82 × 10^−4^	2.68 × 10^−5^	2.69 × 10^−5^	3.29 × 10^−5^	-	5.90 × 10^−4^	1.08 × 10^−4^
		Charcoal Guazuma ulmifolia	2.08 × 10^−4^	2.38 × 10^−2^	5.65 × 10^−4^	5.56 × 10^−5^	6.14 × 10^−5^	1.22 × 10^−5^	-	1.25 × 10^−3^	1.42 × 10^−4^
Dermal	Pork	Wood Eucalyptus citriodora	2.99 × 10^−6^	5.26 × 10^−4^	-	6.12 × 10^−6^	4.98 × 10^−6^	1.76 × 10^−4^	-	4.40 × 10^−3^	2.48 × 10^−5^
		Wood Guazuma ulmifolia	1.03 × 10^−5^	7.14 × 10^−4^	4.07 × 10^−3^	7.01 × 10^−6^	1.17 × 10^−5^	6.19 × 10^−5^	-	7.46 × 10^−3^	4.00 × 10^−5^
		Wood Anadenanthera falcata	1.80 × 10^−5^	3.75 × 10^−4^	4.17 × 10^−3^	5.13 × 10^−6^	1.23 × 10^−5^	-	-	4.70 × 10^−3^	1.94 × 10^−5^
		Wood CCA-Treated Eucalyptus	1.47 × 10^−5^	2.03 × 10^−2^	1.71 × 10^−2^	2.89 × 10^−5^	8.50 × 10^−6^	-	-	6.41 × 10^−3^	2.87 × 10^−5^
		Charcoal Eucalyptus citriodora	1.02 × 10^−5^	1.86 × 10^−3^	4.28 × 10^−3^	7.52 × 10^−6^	7.53 × 10^−6^	2.30 × 10^−4^	-	6.36 × 10^−3^	3.03 × 10^−5^
		Charcoal Guazuma ulmifolia	5.82 × 10^−5^	2.00 × 10^−3^	6.32 × 10^−3^	1.56 × 10^−5^	1.72 × 10^−5^	8.52 × 10^−5^	-	1.34 × 10^−2^	3.97 × 10^−5^
Inhalation	Pork	Wood Eucalyptus citriodora	8.95 × 10^−8^	5.24 × 10^−6^	-	-	-	2.95 × 10^−6^	-	8.57 × 10^−7^	-
		Wood Guazuma ulmifolia	3.07 × 10^−7^	7.13 × 10^−6^	4.56x 10^−7^	-	-	1.04 × 10^−6^	-	1.45 × 10^−6^	-
		Wood Anadenanthera falcata	5.39 × 10^−7^	3.74 × 10^−6^	4.68 × 10^−7^	-	-	-	-	9.14 × 10^−7^	-
		Wood CCA-Treated Eucalyptus	4.39 × 10^−7^	2.02 × 10^−4^	1.92 × 10^−6^	-	-	-	-	1.25 × 10^−6^	-
		Charcoal Eucalyptus citriodora	3.05 × 10^−7^	1.85 × 10^−5^	4.81 × 10^−7^	-	-	3.86 × 10^−6^	-	1.24 × 10^−6^	-
		Charcoal Guazuma ulmifolia	1.74 × 10^−6^	1.99 × 10^−5^	7.10 × 10^−7^	-	-	1.43 × 10^−6^	-	2.61 × 10^−6^	-

**Table 4:** Hazard quotient (HQ) values for Al, As, Cr, Cu, Fe, Mn, Mo, V, and Zn in adults exposed to smoke via ingestion, dermal contact, and inhalation.

HQ for Adults	Meat Type	Smoke Samples	Elements								
			Al	As	Cr	Cu	Fe	Mn	Mo	V	Zn
Ingestion	Beef	Wood Eucalyptus citriodora	1.23 × 10^−6^	1.32 × 10^−3^	-	2.69 × 10^−5^	3.82 × 10^−6^	4.28 × 10^−6^	1.13 × 10^−4^	7.27 × 10^−5^	2.97 × 10^−5^
		Wood Guazuma ulmifolia	3.15 × 10^−6^	1.38 × 10^−4^	-	1.86 × 10^−5^	6.07 × 10^−7^	8.17 × 10^−7^	8.77 × 10^−5^	4.07 × 10^−5^	1.21 × 10^−5^
		Wood Anadenanthera falcata	7.81 × 10^−6^	3.18 × 10^−4^	-	1.68 × 10^−5^	2.27 × 10^−6^	1.63 × 10^−6^	8.49 × 10^−5^	4.61 × 10^−5^	2.13 × 10^−5^
		Wood CCA-Treated Eucalyptus	5.21 × 10^−6^	3.31 × 10^−2^	1.71 × 10^−4^	2.18 × 10^−5^	2.47 × 10^−5^	1.18 × 10^−6^	-	6.35 × 10^−5^	8.65 × 10^−5^
		Charcoal Eucalyptus citriodora	3.23 × 10^−6^	2.13 × 10^−3^	5.19 × 10^−6^	1.22 × 10^−5^	3.31 × 10^−5^	3.52 × 10^−6^	-	6.49 × 10^−5^	8.49 × 10^−5^
		Charcoal Guazuma ulmifolia	1.53 × 10^−5^	7.59 × 10^−4^	1.20 × 10^−4^	1.25 × 10^−5^	3.56 × 10^−5^	1.72 × 10^−6^	-	9.61 × 10^−5^	1.27 × 10^−4^
Dermal	Beef	Wood Eucalyptus citriodora	1.41 × 10^−6^	4.53 × 10^−4^	-	3.07 × 10^−5^	4.35 × 10^−6^	1.22 × 10^−4^	1.29 × 10^−4^	3.19 × 10^−3^	3.38 × 10^−5^
		Wood Guazuma ulmifolia	3.59 × 10^−6^	4.72 × 10^−5^	-	2.12 × 10^−5^	6.92 × 10^−7^	2.33 × 10^−5^	1.00 × 10^−4^	1.78 × 10^−3^	1.38 × 10^−5^
		Wood Anadenanthera falcata	8.91 × 10^−6^	1.09 × 10^−4^	-	1.91 × 10^−5^	2.58 × 10^−6^	4.65 × 10^−5^	9.68 × 10^−5^	2.02 × 10^−3^	2.43 × 10^−5^
		Wood CCA-Treated Eucalyptus	5.93 × 10^−6^	1.13 × 10^−2^	7.79 × 10^−3^	2.49 × 10^−5^	2.82 × 10^−5^	3.36 × 10^−5^	-	2.79 × 10^−3^	9.87 × 10^−5^
		Charcoal Eucalyptus citriodora	3.68 × 10^−6^	7.28 × 10^−4^	2.37 × 10^−4^	1.39 × 10^−5^	3.78 × 10^−5^	1.00 × 10^−4^	-	2.85 × 10^−3^	9.68 × 10^−5^
		Charcoal Guazuma ulmifolia	1.75 × 10^−5^	2.59 × 10^−4^	5.47 × 10^−3^	1.42 × 10^−5^	4.06 × 10^−5^	4.91 × 10^−5^	-	4.21 × 10^−3^	1.45 × 10^−4^
Inhalation	Beef	Wood Eucalyptus citriodora	5.45 × 10^−8^	5.84 × 10^−6^	-	-	-	2.64 × 10^−6^	6.25 × 10^−8^	8.02 × 10^−7^	-
		Wood Guazuma ulmifolia	1.39 × 10^−7^	6.08 × 10^−7^	-	-	-	5.05 × 10^−7^	4.84 × 10^−8^	4.49 × 10^−7^	-
		Wood Anadenanthera falcata	3.45 × 10^−7^	1.41 × 10^−6^	-	-	-	1.01 × 10^−6^	4.68 × 10^−8^	5.08 × 10^−7^	-
		Wood CCA-Treated Eucalyptus	2.30 × 10^−7^	1.46 × 10^−4^	1.13 × 10^−6^	-	-	7.28 × 10^−7^	-	7.01 × 10^−7^	-
		Charcoal Eucalyptus citriodora	1.42 × 10^−7^	9.39 × 10^−6^	3.44 × 10^−8^	-	-	2.18 × 10^−6^	-	7.16 × 10^−7^	-
		Charcoal Guazuma ulmifolia	6.77 × 10^−7^	3.35 × 10^−6^	7.94 × 10^−7^	-	-	1.06 × 10^−6^	-	1.06 × 10^−6^	-
Ingestion	Pork	Wood Eucalyptus citriodora	1.14 × 10^−6^	6.70 × 10^−4^	1.14 × 10^−6^	6.70 × 10^−4^	1.14 × 10^−6^	2.70 × 10^−6^	-	4.38 × 10^−5^	9.48 × 10^−6^
		Wood Guazuma ulmifolia	3.93 × 10^−6^	9.11 × 10^−4^	3.93 × 10^−6^	9.11 × 10^−4^	3.93 × 10^−6^	9.48 × 10^−7^	-	7.42 × 10^−5^	1.53 × 10^−5^
		Wood Anadenanthera falcata	6.90 × 10^−6^	4.78 × 10^−4^	6.90 × 10^−6^	4.78 × 10^−4^	6.90 × 10^−6^	-	-	4.67 × 10^−5^	7.41 × 10^−6^
		Wood CCA-Treated Eucalyptus	5.61 × 10^−6^	2.59 × 10^−2^	5.61 × 10^−6^	2.59 × 10^−2^	5.61 × 10^−6^	-	-	6.38 × 10^−5^	1.10 × 10^−5^
		Charcoal Eucalyptus citriodora	3.90 × 10^−6^	2.37 × 10^−3^	3.90 × 10^−6^	2.37 × 10^−3^	3.90 × 10^−6^	3.53 × 10^−6^	-	6.33 × 10^−5^	1.16 × 10^−5^
		Charcoal Guazuma ulmifolia	2.23 × 10^−5^	2.55 × 10^−3^	2.23 × 10^−5^	2.55 × 10^−3^	2.23 × 10^−5^	1.30 × 10^−6^	-	1.33 × 10^−4^	1.52 × 10^−5^
Dermal	Pork	Wood Eucalyptus citriodora	1.30 × 10^−6^	2.29 × 10^−4^	1.30 × 10^−6^	2.29 × 10^−4^	1.30 × 10^−6^	7.68 × 10^−5^	-	1.92 × 10^−3^	1.08 × 10^−5^
		Wood Guazuma ulmifolia	4.48 × 10^−6^	3.12 × 10^−4^	4.48 × 10^−6^	3.12 × 10^−4^	4.48 × 10^−6^	2.70 × 10^−5^	-	3.26 × 10^−3^	1.75 × 10^−5^
		Wood Anadenanthera falcata	7.86 × 10^−6^	1.64 × 10^−4^	7.86 × 10^−6^	1.64 × 10^−4^	7.86 × 10^−6^	-	-	2.05 × 10^−3^	8.44 × 10^−6^
		Wood CCA-Treated Eucalyptus	6.39 × 10^−6^	8.85 × 10^−3^	6.39 × 10^−6^	8.85 × 10^−3^	6.39 × 10^−6^	-	-	2.80 × 10^−3^	1.25 × 10^−5^
		Charcoal Eucalyptus citriodora	4.44 × 10^−6^	8.09 × 10^−4^	4.44 × 10^−6^	8.09 × 10^−4^	4.44 × 10^−6^	1.00 × 10^−4^	-	2.77 × 10^−3^	1.32 × 10^−5^
		Charcoal Guazuma ulmifolia	2.54 × 10^−5^	8.72 × 10^−4^	2.54 × 10^−5^	8.72 × 10^−4^	2.54 × 10^−5^	3.72 × 10^−5^	-	5.85 × 10^−3^	1.73 × 10^−5^
Inhalation	Pork	Wood Eucalyptus citriodora	5.05 × 10^−8^	2.96 × 10^−6^	-	-	-	1.66 × 10^−6^	-	4.83 × 10^−7^	-
		Wood Guazuma ulmifolia	1.73 × 10^−7^	4.02 × 10^−6^	2.57 × 10^−7^	-	-	5.86 × 10^−7^	-	8.19 × 10^−7^	-
		Wood Anadenanthera falcata	3.04 × 10^−7^	2.11 × 10^−6^	2.64 × 10^−7^	-	-	-	-	5.15 × 10^−7^	-
		Wood CCA-Treated Eucalyptus	2.47 × 10^−7^	1.14 × 10^−4^	1.08 × 10^−6^	-	-	-	-	7.03 × 10^−7^	-
		Charcoal Eucalyptus citriodora	1.72 × 10^−7^	1.04 × 10^−5^	2.71 × 10^−7^	-	-	2.18 × 10^−6^	-	6.98 × 10^−7^	-
		Charcoal Guazuma ulmifolia	9.83 × 10^−7^	1.12 × 10^−5^	4.00 × 10^−7^	-	-	8.05 × 10^−7^	-	1.47 × 10^−6^	-

**Table 5:** HI values for non-carcinogenic risks and HIt (integrated per exposure pathway and total aggregate).

			Wood				Charcoal	
	Exposure Pathway	Sample	*Eucalyptus citriodora*	*Guazuma ulmifolia*	*Anadenanthera* *falcata*	CCA-Treated Eucalyptus	*Eucalyptus* *citriodora*	*Guazuma* *ulmifolia*
Children	HI Inh	Beef	1.67 × 10^−5^	3.10 × 10^−6^	5.87 × 10^−6^	2.64 × 10^−4^	2.21 × 10^−5^	1.23 × 10^−5^
	HI Ing		1.47 × 10^−2^	2.81 × 10^−3^	4.66 × 10^−3^	3.13 × 10^−1^	2.18 × 10^−2^	1.09 × 10^−2^
	HI Der		9.08 × 10^−3^	4.57 × 10^−3^	5.33 × 10^−3^	5.07E x 10^−2^	9.32 × 10^−3^	2.34 × 10^−2^
	HIt		0.024	0.007	0.010	0.364	0.031	0.034
Adult	HI Inh		9.40 × 10^−6^	1.75 × 10^−6^	3.31 × 10^−6^	1.49 × 10^−4^	1.25 × 10^−5^	6.94 × 10^−6^
	HI Ing		1.58 × 10^−3^	3.02 × 10^−4^	4.99 × 10^−4^	3.35 × 10^−2^	2.33 × 10^−3^	1.17 × 10^−3^
	HI Der		3.96 × 10^−3^	1.99 × 10^−3^	2.33 × 10^−3^	2.21 × 10^−2^	4.06 × 10^−3^	1.02 × 10^−2^
	HIt		0.006	0.002	0.003	0.056	0.006	0.011
Children	HI Inh	Pork	9.14 × 10^−6^	1.04 × 10^−5^	5.66 × 10^−6^	2.06 × 10^−4^	2.44 × 10^−5^	2.64 × 10^−5^
	HI Ing		6.83 × 10^−3^	9.82 × 10^−3^	5.47 × 10^−3^	2.44 × 10^−1^	2.33 × 10^−2^	2.60 × 10^−2^
	HI Der		5.14 × 10^−3^	1.24 × 10^−2^	9.29 × 10^−3^	4.38 × 10^−2^	1.28 × 10^−2^	2.12 × 10^−2^
	Hit		0.012	0.022	0.015	0.288	0.036	0.048
Adult	HI Inh		5.16 × 10^−6^	5.86 × 10^−6^	3.19 × 10^−6^	1.16 × 10^−4^	1.38 × 10^−5^	1.49 × 10^−5^
	HI Ing		7.32 × 10^−4^	1.05 × 10^−3^	5.86 × 10^−4^	2.61 × 10^−2^	2.50 × 10^−3^	2.80 × 10^−3^
	HI Der		2.24 × 10^−3^	5.40 × 10^−3^	4.05 × 10^−3^	1.91 × 10^−2^	5.58 × 10^−3^	9.58 × 10^−3^
	Hit		0.003	0.006	0.005	0.045	0.008	0.012

**Table 6:** Carcinogenic risks of each element for children and adults through inhalation, ingestion, and dermal exposure.

Carcinogenic Risk (CR)				Wood				Charcoal	
				*Eucalyptus* *citriodora*	*Guazuma* *ulmifolia*	*Anadenanthera falcata*	CCA-Treated *Eucalyptus*	*Eucalyptus citriodora*	*Guazuma* *ulmifolia*
Beef	Ingestion	As	Children	4.76 × 10^−7^	4.96 × 10^−8^	1.15 × 10^−7^	1.19 × 10^−5^	7.66 × 10^−7^	2.73 × 10^−7^
			Adult	3.95 × 10^−5^	2.16 × 10^−5^	2.45 × 10^−5^	3.16 × 10^−5^	3.55 × 10^−5^	4.45 × 10^−5^
		Cr	Children	-	-	-	2.05 × 10^−7^	6.23 × 10^−9^	1.44 × 10^−7^
			Adult	-	-	-	8.79 × 10^−8^	2.67 × 10^−9^	6.17 × 10^−8^
	Dermal	As	Children	4.00 × 10^−8^	4.17 × 10^−9^	9.63 × 10^−9^	1.00 × 10^−6^	6.43 × 10^−8^	2.29 × 10^−8^
			Adult	1.35 × 10^−5^	7.40 × 10^−6^	8.37 × 10^−6^	1.08 × 10^−5^	1.21 × 10^−3^	1.52 × 10^−5^
		Cr	Children	-	-	-	2.30 × 10^−6^	6.98 × 10^−8^	1.61 × 10^−6^
			Adult	-	-	-	4.01 × 10^−6^	1.22 × 10^−7^	2.82 × 10^−6^
	Inhalation	As	Children	2.00 × 10^−11^	2.08 × 10^−12^	4.81 × 10^−12^	5.00 × 10^−10^	3.21 × 10^−11^	1.14 × 10^−11^
			Adult	4.50 × 10^−11^	4.69 × 10^−12^	1.08 × 10^−11^	1.13 × 10^−9^	7.24 × 10^−11^	2.58 × 10^−11^
		Cr	Children	-	-	-	8.59 × 10^−12^	2.61 × 10^−13^	6.04 × 10^−12^
			Adult	-	-	-	1.94 × 10^−11^	5.89 × 10^−13^	1.36 × 10^−11^
Pork	Ingestion	As	Children	2.41 × 10^−7^	3.28 × 10^−7^	1.72 × 10^−7^	9.31 × 10^−6^	8.52 × 10^−7^	9.18 × 10^−7^
			Adult	2.20 × 10^−5^	3.73 × 10^−5^	2.44 × 10^−5^	3.21 × 10^−5^	3.45 × 10^−5^	4.17 × 10^−5^
		Cr	Children	-	4.67 × 10^−8^	4.78 × 10^−8^	1.96 × 10^−7^	4.92 × 10^−8^	7.26 × 10^−8^
			Adult	-	2.00 × 10^−8^	2.05 × 10^−8^	8.39 × 10^−8^	2.11 × 10^−8^	3.11 × 10^−8^
	Dermal	As	Children	2.03 × 10^−8^	2.76 × 10^−8^	1.45 × 10^−8^	7.82 × 10^−7^	7.16 × 10^−8^	7.71 × 10^−8^
			Adult	7.52 × 10^−6^	1.28 × 10^−5^	8.33 × 10^−6^	1.10 × 10^−5^	1.18 × 10^−5^	1.43 × 10^−5^
		Cr	Children	-	5.23 × 10^−7^	5.36 × 10^−7^	2.19 × 10^−6^	5.51 × 10^−7^	8.13 × 10^−7^
			Adult	-	9.12 × 10^−7^	9.35 × 10^−7^	3.83 × 10^−6^	9.61 × 10^−7^	1.42 × 10^−6^
	Inhalation	As	Children	1.01 × 10^−11^	1.37 × 10^−11^	7.22 × 10^−12^	3.90 × 10^−10^	3.57 × 10^−11^	3.85 × 10^−11^
			Adult	2.28 × 10^−11^	3.10 × 10^−11^	1.63 × 10^−11^	8.80 × 10^−10^	8.05 × 10^−11^	8.68 × 10^−11^
		Cr	Children	-	1.96 × 10^−12^	2.01 × 10^−12^	8.21 × 10^−12^	2.06 × 10^−12^	3.04 × 10^−12^
			Adult	-	4.41 × 10^−12^	4.52 × 10^−12^	1.85 × 10^−11^	4.65 × 10^−12^	6.86 × 10^−12^

## Data Availability

Data are available upon reasonable request to the corresponding author.
